# TaTypA, a Ribosome-Binding GTPase Protein, Positively Regulates Wheat Resistance to the Stripe Rust Fungus

**DOI:** 10.3389/fpls.2016.00873

**Published:** 2016-06-21

**Authors:** Peng Liu, Thwin Myo, Wei Ma, Dingyun Lan, Tuo Qi, Jia Guo, Ping Song, Jun Guo, Zhensheng Kang

**Affiliations:** State Key Laboratory of Crop Stress Biology for Arid Areas, College of Plant Protection, Northwest A&F UniversityYangling, China

**Keywords:** TypA, *Puccinia striiformis* f. sp. *tritici*, ROS, virus-induced gene silencing, *Triticum aestivum*, tyrosine phosphorylation

## Abstract

Tyrosine phosphorylation protein A (TypA/BipA) belongs to the ribosome-binding GTPase superfamily. In many bacterial species, TypA acts as a global stress and virulence regulator and also mediates resistance to the antimicrobial peptide bactericidal permeability-increasing protein. However, the function of *TypA* in plants under biotic stresses is not known. In this study, we isolated and functionally characterized a stress-responsive *TypA* gene (*TaTypA*) from wheat, with three copies located on chromosomes 6A, 6B, and 6D, respectively. Transient expression assays indicated chloroplast localization of TaTypA. The transcript levels of *TaTypA* were up-regulated in response to treatment with methyl viologen, which induces reactive oxygen species (ROS) in chloroplasts through photoreaction, cold stress, and infection by an avirulent strain of the stripe rust pathogen. Knock down of the expression of *TaTypA* through virus-induced gene silencing decreased the resistance of wheat to stripe rust accompanied by weakened ROS accumulation and hypersensitive response, an increase in *TaCAT* and *TaSOD* expression, and an increase in pathogen hyphal growth and branching. Our findings suggest that *TaTypA* contributes to resistance in an ROS-dependent manner.

## Introduction

During the growth of plants that are subjected to different environmental stresses, including drought, salinity, chilling, and metal toxicity as well as pathogen attack. In these biotic and abiotic stresses, reactive oxygen species (ROS) are generated in plants due to a disruption of cellular homeostasis (Sharma and Dubey, 2005; Srivastava and Dubey, 2011). ROS are comprised of free radicals, such as superoxide anion (O_2_^⋅-^) and hydroxyl radical (^⋅^OH), as well as non-radical molecules, like hydrogen peroxide (H_2_O_2_) and singlet oxygen (^1^O_2_; Sharma and Dubey, 2005). Diverse mechanisms have been shown to be involved in pathogen-induced ROS production, including peroxidases and different oxidases (oxalate oxidase, amine oxidase, and NADPH oxidase; Pugin et al., 1997; Wojtaszek, 1997; Rea et al., 1998). The increased production of ROS during biotic and abiotic stresses can pose a threat to plant cells by causing oxidation of proteins, peroxidation of lipids, damage to nucleic acids, inhibition of enzymes, activation of the programmed cell death (PCD) pathway, and ultimately lead to death of the cells. ROS act as more likely cofactors in redox reactions taking part in various roles in plant defenses (Torres, 2010). For example, ROS have been characterized as primary signaling molecules modulating multiple physiological processes during plant growth and development (De Tullio, 2010). Intriguingly, evolutionary considerations based on the NADPH gene family imply that mechanisms detoxifying ROS were acquired before the plants used ROS as signaling molecules (Mittler et al., 2011). The reasons that make ROS important signaling regulators are: (i) rapid control over the production and scavenging of ROS in individual cells, allowing a dynamic control of ROS levels; (ii) accumulation of ROS in different subcellular organelles, resulting in an efficient intracellular control; (iii) rapid propagation of ROS-induced signaling from the origin of the stimuli to nearby cells; (iv) interaction and modification of a variety of targets by ROS (Mittler et al., 2011). Remarkably, plants exploit this versatility of ROS when responding to the environment and during biotic interactions (Scheler et al., 2013).

Tyrosine phosphorylation protein A (TypA/BipA) is a member of the ribosome-binding GTPase superfamily. GTPases are widely distributed molecular switches found across all bacterial species (Robinson, 2008) and are involved in the regulation of multiple cellular processes, including protein translocation, translation, tRNA modification, ribosome biogenesis and assembly, cell polarity, cell division and diverse signaling events (Verstraeten et al., 2011). Translational GTPases (trGTPases) are involved in GTPase activity that is induced by the large ribosomal subunit of bacterial species (Ramakrishnan, 2002; Nilsson and Nissen, 2005; Margus et al., 2007). A few members of trGTPases family, such as EF-G, EF-Tu, IF2 and RF3, bind to an overlapping site on the ribosome (Sergiev et al., 2005). TypA/BipA was firstly identified in *Salmonella typhimurium* as a protein induced by the antimicrobial peptide bactericidal permeability-increasing protein (BPI; Qi et al., 1995). Previous studies suggested that TypA has an effect on expression of the global regulator Fis by destabilizing unusually strong interactions between the 5′-untranslated region of *fis* mRNA and the ribosome. TypA binding to ribosomes at a site in accordance with that of EF-G indicated that GTPase activity of TypA is sensitive to high GDP:GTP ratios (Owens et al., 2004). Several studies showed that TypA is involved in regulating diverse virulence-related mechanisms in *Escherichia coli*, including flagella-mediated cell motility and cytoskeletal rearrangements in host epithelial cells (Farris et al., 1998), capsule synthesis and expression of genes from different pathogenicity islands (Grant et al., 2003). TypA is necessary for survival of *Sinorhizobium meliloti* under some stress conditions, such as low pH, low temperature, and treatment with sodium dodecyl sulfate (SDS; Kiss et al., 2004) and is required for growth of *E. coli* K12 at low temperatures (Pfennig and Flower, 2001). TypA are widely distributed in plants and are present in green algae, mosses, liverworts, gymnosperms, and angiosperms (Barak and Trebitsh, 2007). Recently, Atkinson (2015) identified a previously unreported TypA subfamily (mTypA) which are mitochondrially targeted according to transit peptide predictions in some Archaeplastida, Amoebozoa, and fungi (Atkinson, 2015). *TypA* genes also have roles in pollen tube growth in *Arabidopsis* (Lalanne et al., 2004) and in development of male reproductive organs in cucumber (Barak and Trebitsh, 2007). SsTypA was proposed as a new member of the ROS-scavenging system in the chloroplast of *Suaeda salsa* when subjected to environmental stress (Wang et al., 2008). However, little is known about the functions of *TypA* gene in plant defense against pathogens.

Wheat stripe rust, caused by *Puccinia striiformis* f. sp. *tritici* (*Pst*), is a destructive disease of wheat (*Triticum aestivum*) worldwide (Hau and de Vallavieille-Pope, 2006). The fungus is a strict biotroph and is dependent on living host cells for growth and survival. This life style has hindered study of the molecular mechanisms involved in the wheat-*Pst* interaction. In a previous study, to isolate defense-related genes against the fungus, we constructed an incompatible suppression subtractive hybridization (SSH) cDNA library of wheat leaves infected by *Pst* and identified a cDNA fragment exhibiting high homology to the rice TypA protein (Yu et al., 2010). In the present work, we identified a wheat *TypA* homolog, designated *TaTypA*. The transcript profile of *TaTypA* was analyzed in wheat seedlings inoculated with *Pst* and in plants subjected to environmental stimuli, and the subcellular localization of *TaTypA* was determined. Furthermore, knock down of *TaTypA* in wheat was performed to analyze whether and how *TaTypA* participates in resistance to stripe rust. Our results demonstrated that *TaTypA* performs a positive function in resistance by regulating ROS-induced signaling.

## Materials and Methods

### Plant Materials, Fungal Pathogens, and Treatments

Wheat cv. Suwon 11 (Su11) and isolates of *Pst* pathotypes CYR23 (avirulent) and CYR31 (virulent) were used in the study. Su11, carrying the *YrSu* resistance gene, expresses a typical hypersensitive response (HR) to CYR23, but is susceptible to CYR31 (Cao et al., 2002). Wheat seedlings were grown, inoculated and maintained following procedures and conditions described previously (Kang et al., 2002). For RNA isolation wheat leaves were collected at 0, 12, 24, 36, 48, 72, 96, and 120 h post-inoculation (hpi) with CRY23 or CYR31. The time points were selected based on a microscopic study of the interactions between Su11 with CYR23 and CYR31 (Kang et al., 2002; Wang et al., 2007). A methyl viologen (MV) concentration gradient assay was conducted to induce oxidative stress, whereby 10-days-old wheat seedlings were sprayed with 0.1, 1, or 5 mM MV. Samples were collected at 0, 4, 6, 8, 12, and 24 h hpt. Control wheat seedlings were treated with sterile distilled water in both treatments. Ten-days-old wheat seedlings were placed in a 4°C chamber for low-temperature treatment. Roots of 10-days-old wheat seedlings were soaked in 20% PEG 6000 for the drought stress treatment, or in 200 mM NaCl to induce salt stress. The first leaves of the treated and control plants treated with sterile distilled water were collected at 0, 6, 12, and 24 hpt. Three biological replicates were included in each assay. Tissues harvested from all treatments were immediately frozen in liquid nitrogen and stored at -80°C until the extraction of total RNA.

### Identification and Sequence Analysis of the *TaTypA* Gene

A 433-bp unisequence (GenBank accession number EV253998) exhibiting high homology to a putative TypA *Oryza sativa* GTP-binding protein was obtained from the *Pst*-induced SSH cDNA library (Yu et al., 2010) and used as a query sequence to screen EST databases of wheat constructed in our laboratory (Ma et al., 2009). Homologous wheat EST clones were retrieved and assembled, and according to the assembled sequence, we designed the TaTypA-F and TaTypA-R primers (**Supplementary Table S1**) to amplify *TaTypA*. The amplified product was cloned into the pGEM-T Easy Vector (Promega, Madison, WI, USA) for sequencing. This cDNA product was aligned with the wheat cv. Chinese Spring (CS) genome using data of the International Wheat Genome Sequencing Consortium^1^. The predicted chromosomal location and related sequences were also obtained from this website. The cDNA sequences were analyzed with ORF Finder^2^ and the BLAST^3^ programs. The amino acid sequence was analyzed with Pfam^4^, InterProScan^5^ and PROSITE Scan^6^ to identify conserved domains. Multiple sequence alignment was performed with ClustalW2.0 (Chenna et al., 2003) and DNAMAN7.0 software (Lynnon Biosoft, USA), and a phylogenetic tree of alignment of amino acid sequences was generated with Mega 5.0 software (Tamura et al., 2011).

### RNA Extraction, cDNA Synthesis, and qRT-PCR

Total RNA was extracted with the Trizol^TM^ Reagent (Invitrogen, Carlsbad, CA, USA) following the manufacturer’s instructions. Three μg of RNA were subjected to first strand cDNA synthesis with the Promega RT-PCR system (Promega, Madison, WI, USA) and Oligo (dT) 18 primer. Relative quantification of *TaTypA* expression was performed with a SYBR Green qRT-PCR mixture on an ABI prism 7500 sequence detection system (Applied Biosystems, USA). Specific primers (**Supplementary Table S1**) were designed and qRT-PCR was conducted according to previously described procedures (Wang et al., 2009). The wheat translation elongation factor *TaEF-1α* (GenBank Accession number Q03033) was used as an internal reference for normalization. Dissociation curves were produced for each reaction to ensure specific amplification. Threshold Ct values were generated from the ABI PRISM 7500 Software Tool (Applied Biosystems) to quantify relative gene expression. Transcript levels of *TaTypA* were calculated by the comparative 2^-ΔΔCT^ method (Livak and Schmittgen, 2001). Transcript abundance was assessed from three independent biological replicates.

### Subcellular Location of TaTypA-GFP Fusion Protein and Western Blotting

Protoplasts for subcellular localization of TaTypA protein were isolated from mesophyll tissue of 1-week-old wheat seedlings as previously described (Li et al., 2015). A recombinant plasmid, pCaMV35S::TaTypA::GFP, was constructed by cloning full-length cDNA and of *TaTypA* into a pCaMV35S::GFP vector by PCR using primers TaTyPA-163-F and TaTyPA-163-R (**Supplementary Table S1**). For analysis of the chloroplast transit peptide in TaTypA, the partial coding sequence (positions 1–240, 240 nt) at N-terminal was fused into pCaMV35S::GFP vector by PCR using primers TaTyPA-163-F and TaTyPA-163-R_N_ (**Supplementary Table S1**; pCaMV35S::TaTypA-N_1-80_::GFP). Wheat mesophyll protoplasts were then transformed by the polyethylene glycol (PEG) transfection method using the plasmid DNA of pCaMV35S::TaTypA::GFP and pCaMV35S::TaTypA-N_1-80_::GFP, pCaMV35S::GFP as the control. The PEG-transfected mesophyll protoplasts were incubated in W5 solution in a dark chamber at 23°C for 18 h, and GFP fluorescence was observed under a confocal laser scanning microscope (Zeiss LSM 700, Germany) as previously described (Ito and Shinozaki, 2002).

The total protein in the protoplast pellet was extracted with protein extraction kits (Solarbio, Beijing, China) following the manufacturer’s instructions. Western blotting assays were performed using the total protein by 12% SDS-PAGE. After the proteins were transferred to nitrocellulose membranes (Millipore), the membranes were incubated in blocking buffer (0.05% Tween 20 and 5% non-fat milk powder in TBS). GFPs were detected using mouse-derived GFP-antibodies (Sungene, Tianjing, China) diluted in blocking buffer at 1:5000 overnight at 4°C. Membranes were washed and incubated with horseradish peroxidase-conjugated antimouse secondary antibody (Sungene, Tianjing, China) diluted at 1:10,000 and chemiluminescence substrate for detection (Sigma, Tokyo, Japan).

### BSMV-Mediated TaTypA Gene Silencing

cDNA fragments of *TaTypA* with *Not*I and *Pac*I restriction sites were obtained by reverse transcription PCR to modify the original BSMV:γ vector for gene silencing as previously described (Holzberg et al., 2002). The fragments show no similarity with any other wheat gene in BLAST analyses, indicating their specificity. Capped *in vitro* transcripts of BSMV RNAs were prepared from the linearized plasmids (Petty et al., 1990) γ-TaPDS-as, γ-TaTypA-as, γ, α, β using a Message T7 *in vitro* transcription kit (Ambion, Austin, TX, USA) according to the manufacturer’s protocol; 2.5 μl of each transcript, including BSMV RNAs α, β and genetically modified γ, were added to 42.5 μl of FES buffer (0.1 M glycine, 0.06 M K_2_HPO_4_, 1% w/v tetrasodium pyrophosphate, 1% w/v bentonite, and 1% w/v celite, pH 8.5) and inoculated into the second leaves of two-leaf wheat seedlings by gentle rubbing of the surface with a gloved finger (Scofield et al., 2005). After 24 h of incubation in a dark, humid environment, the seedlings were maintained in a growth chamber at 25°C with a 16 h photoperiod, and virus symptoms were monitored at regular intervals. Recombinant virus BSMV:TaPDS-as was used as a positive control in all experiments. Mock-treated plants were inoculated with 1× Fes buffer as a negative control. When virus symptoms were observed at 10 days post-viral inoculation, symptom phenotypes were photographed, and the fourth seedling leaves were inoculated with fresh urediniospores of *Pst* pathotypes CYR23 or CYR31. For RNA isolation and histological observation, leaves were sampled at 0, 24, 48, and 120 hpi with CYR23. The *Pst* infection phenotypes were also photographed at 15 dpi. This experiment was conducted with three independent biological replications.

### Histology of Fungal Growth and Host Response

Wheat leaves infected with BSMV were sampled at 24, 48, and 120 hpi with *Pst* and stained as described (Wang et al., 2007). The fourth leaves pre-infected with BSMV were sampled at 24, 48, and 120 hpi with *Pst* race CYR23. The areas of necrosis in infected leaves were estimated by auto-fluorescence of mesophyll cells. Hyphal length and hyphal branches were examined under blue light excitation by epi-fluorescence microscopy (excitation filter, 485 nm; dichromic mirror, 510 nm; and barrier filter, 520 nm). H_2_O_2_ accumulation was detected by staining with 3,3′-diaminobenzidine (DAB; Amresco, Solon, OH, USA) as previously described (Wang et al., 2007), then viewed by differential interference contrast optics. Only a site where an appressorium had formed over a stoma was considered to be successfully penetrated. At least 50 infection sites were examined on each of five randomly selected leaf segments for each treatment. Necrotic areas, areas of H_2_O_2_ accumulation, and hyphal length were observed with an Olympus BX-53 microscope (Olympus, Corp., Tokyo) and estimated with DP2-TWAIN/DP2-BSW software. Statistical analysis was performed by Tukey’s HSD test (*P* < 0.05) with the use of SPSS software (SPSS, Inc., Chicago, IL, USA).

## Results

### Cloning and Structural Features of *TaTypA*

A wheat 2,278-bp homolog of tyrosine phosphorylation protein A, first identified *in silico*, was obtained. It had an open reading frame (ORF) of 2,028 bp and was designated as *TaTypA* (GenBank accession number KF309066). Sequence alignment in the Chinese Spring genome database indicated that each sub-genome (A, B, or D) contained more than three sequences, each lacking the full *TaTypA* coding sequence likely due to incomplete Chinese Spring genome sequences. However, all sub-genomic sequences were localized on the long arm of chromosomes 6A, 6B, and 6D (data not shown). To better understand *TaTypA* characteristics in the wheat genome, the exon of each sub-genomic sequence was selected and assembled artificially, designated *TaTypA-6A, TaTypA-6B*, and *TaTypA-6D*, respectively (**Supplementary Figure S1**). The *TaTypA* gene from wheat cv. Su11 exhibited 98.67, 77.78 and 98.52% identities with the sequences from *TaTypA-6A, TaTypA-6B*, and *TaTypA-6D*, respectively (**Supplementary Figure S1**). TaTypA-6A and TaTypA-6D contain only a few amino acid variations relative to TaTypA from Su11, whereas TaTypA-6B contains 101- and 48-aa deletions (**Supplementary Figure S2**). We concluded that the wheat genome contains three homologous copies of *TaTypA* located on the long arms of chromosomes 6A, 6B, and 6D.

Sequence analysis indicated that *TaTypA* encodes a putative protein composed of 675 amino acid residues, a molecular weight of 74.25 kDa, and an isoeletric point (pI) of 5.65. Multi-sequence alignment with TypA from other higher plants showed that Su11 TaTypA shares highest identity (94.09%) with TypA from *Brachypodium distachyon.* In addition, TaTypA shared 89, 75, and 74% identity with OsTypA, SsTypA1, and AtTypA, respectively (**Supplementary Figure S3**). Structural analysis showed that TaTypA has three conserved regions, including an ATP/GTP-binding motif, a GTP-binding elongation factor signature, and a putative ribosome-binding domain (**Supplementary Figure S3**). Conserved tyrosine residues were also present.

**FIGURE 1:**
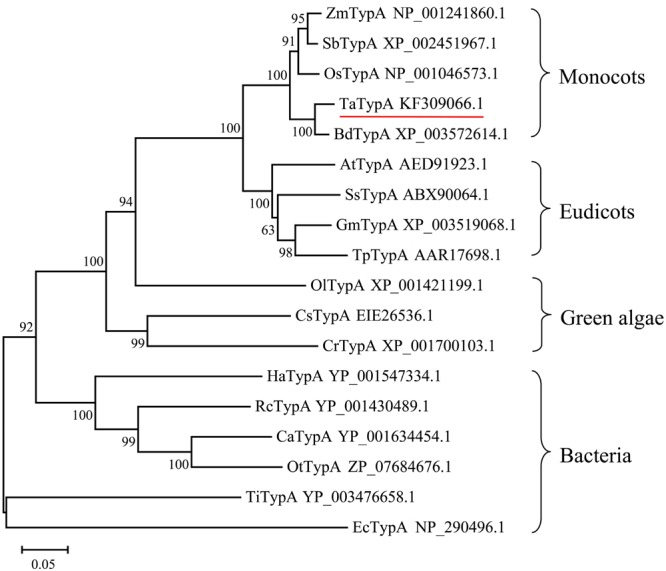
**Neighbor-joining tree of TypA protein sequences based on multiple alignments.** The number above the internal branches indicates bootstrap values estimated based on 1,000 replications. Scale bar represents the estimated number of amino acid substitutions per site. Branches are labeled with protein names and GenBank accession numbers. Ta, *Triticum aestivum*; Bd, *Brachypodium distachyon*; Os, *Oryza sativa*; Zm, *Zea mays*; Sb, *Sorghum bicolor*; Gm, *Glycine max*; Ss, *Suaeda salsa*; At, *Arabidopsis thaliana*; Tp, *Trifolium pretense*; Ol, *Ostreococcus lucimarinus*; Cs, *Coccomyxa subellipsoidea*; Cr, *Chlamydomonas reinhardtii*; Ca, *Chloroflexus aurantiacus*; Rc, *Roseiflexus castenholzii*; Ha, *Herpetosiphon aurantiacus*; Ot, *Oscillochloris trichoides*; Ti, *Thermoanaerobacter italicus*; Ec, *Escherichia coli*.

TypA/BipA proteins from different organisms were selected to study the evolutionary relationships of TypA homologs. The neighbor-joining phylogenetic tree (**Figure 1**) showed that the proteins share a common ancestor with bacterial TypA and can be grouped into four groups. Plant TypA proteins formed a monophyletic group containing monocot and eudicot subgroups. TaTypA, together with OsTypA from *Oryza sativa* and BdTypA from *B. distachyon*, constituted a monocot subgroup. Homologous proteins from dicotyledonous plants clustered into another group. The short branch length (**Figure 1**) indicated that TypA proteins from different organisms share close evolutionary relationships.

### TaTypA Is Mainly Localized in Chloroplasts of Wheat Cells

**FIGURE 2:**
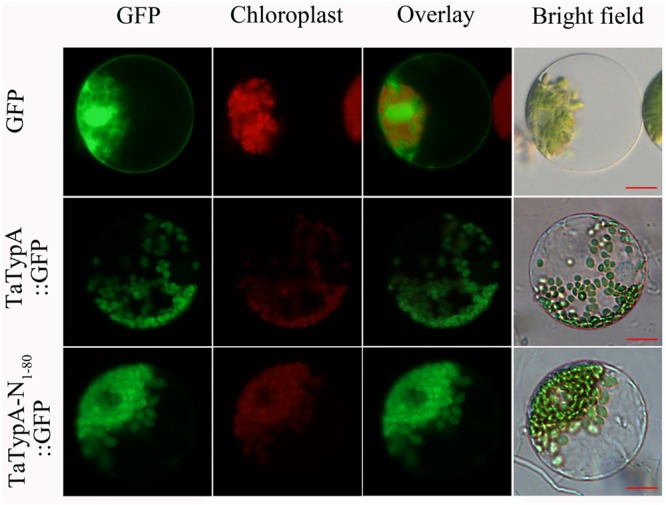
**Subcellular localization of TaTypA protein in wheat protoplasts.** GFP signals are indicated by green color. All images were observed with a fluorescence microscope. Bar, 20 μm.

To determine the subcellular localization of TaTypA in wheat, recombinant pCaMV35S::TaTypA::GFP and pCaMV35S::TaTypA-N_1-80_::GFP were transfected into wheat mesophyll protoplasts by transfection. The empty pCaMV35S::GFP vector was used as a control. GFP was ubiquitously distributed throughout the cell, including in the nucleus (**Figure 2**). pCaMV35S::TaTypA::GFP and pCaMV35S::TaTypA-N_1-80_::GFP fusion proteins were mainly targeted to the chloroplasts of wheat cells (**Figure 2**). To further confirm this result, a Western blot was performed using GFP antibodies on the subcellular fractions of pCaMV35S::GFP, pCaMV35S::TaTypA::GFP, and pCaMV35S::TaTypA-N_1-80_::GFP transfected wheat protoplasts. Bands of 28-, 107-, and 35-kDa were detected, suggesting that GFP, TapA-GFP and TaTypA-N_1-80_-GFP was expressed in wheat cells (**Supplementary Figure S4**). These results from fluorescence microscope and Western blot analysis indicated that TaTypA-GFP was targeted to wheat chloroplast.

### Transcript Profiles of *TaTypA* in Response to Oxidative Stress and Abiotic Stress

**FIGURE 3:**
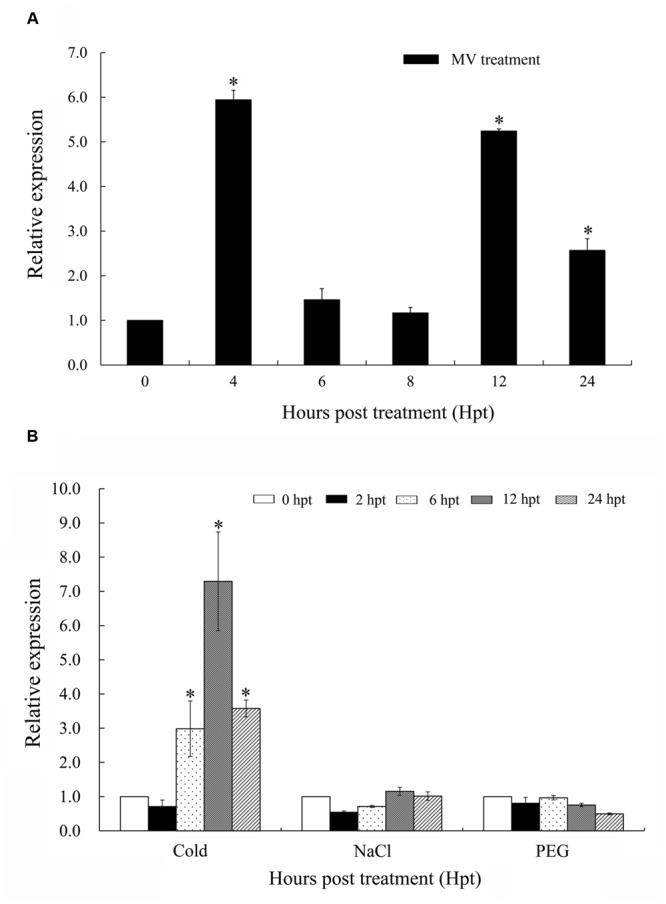
**Expression patterns of *TaTypA* in wheat leaves based on qRT-PCR analyses. (A)** Expression levels of *TaTypA* in wheat leaves in response to MV treatment. **(B)** Expression profiles of TaTypA in wheat leaves after treatment with environmental stresses: low temperature (4°C), salt (NaCl), and drought (PEG). The expression levels were analyzed by qRT-PCR and normalized to the wheat elongation factor *TaEF-1a* gene. Means and standard deviations were based on three independent biological replicates. Asterisks indicate a significant difference (*P* < 0.05) from 0 hpt by Tukey’s HSD test.

When treated with MV, a superoxide anion propagator, the wheat seedlings wilted due to oxidative stress (**Supplementary Figure S5**). Wheat seedlings treated with 5 mM MV rapidly showed damping off symptoms, whereas seedlings treated with 0.1 mM MV were only slightly affected up to 12 hpt. However, seedlings treated with 1 mM MV expressed intermediate symptoms. Therefore, 1 mM MV was selected as a suitable concentration for the oxidative stress treatment. *TaTypA* transcripts were up-regulated and peaked as early as 4 hpt at a level approximately sixfold higher. The transcript level subsequently fell reaching the basal level at 6 and 8 hpt, but increased again at 12 hpt until 24 hpt (**Figure 3A**).

Considering the involvement of *TypA* in response to various abiotic stresses in *S. salsa*, we investigated the effects of various abiotic stresses on expression of *TaTypA*. The transcript level of *TaTypA* increased threefold at 6 h after low-temperature treatment and reached a maximum of about sevenfold higher than the base level at 12 hpt (**Figure 3B**). No significant change (less than twofold, *P* > 0.05) in *TaTypA* transcript was detected in wheat leaves after treatment with salt or PEG (**Figure 3B**).

### Transcript Analysis of *TaTypA* in Response to *Pst*

**FIGURE 4:**
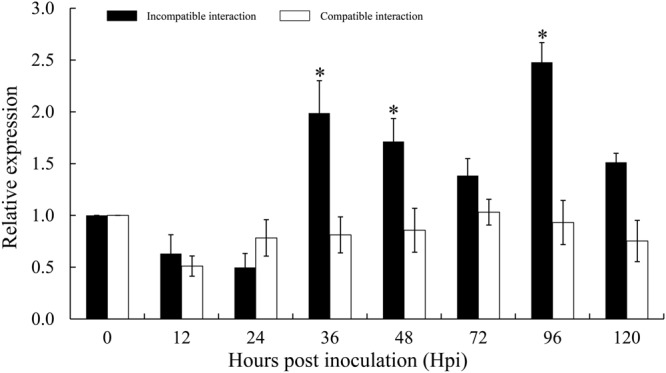
**Expression profiles of *TaTypA* in wheat leaves in response to virulent and avirulent *Pst* races.** Wheat leaves inoculated with *Pst* isolates CYR23 (avirulent) and CYR31 (virulent) were sampled at 0, 6, 12, 24, 48, and 120 h post-inoculation. The data were normalized to the wheat *TaEF-1a* gene, and error bars represent the variation among three independent replicates. Asterisks indicate significant differences from 0 hpi using Tukey’s HSD test (*P* < 0.05).

To investigate whether *TaTypA* is involved in response to *Pst*, quantitative RT-PCR was used to analyze *TaTypA* transcript profiles in both compatible and incompatible interactions. In the incompatible combination, transcript levels were induced at 36 hpi and reached the highest level of 2.5-fold higher that in control plants at 96 hpi. In the compatible combination there was no significant change in relative *TaTypA* expression (**Figure 4**).

### Suppression of *TaTypA* Enhances Susceptibility of Wheat to *Pst*

**FIGURE 5:**
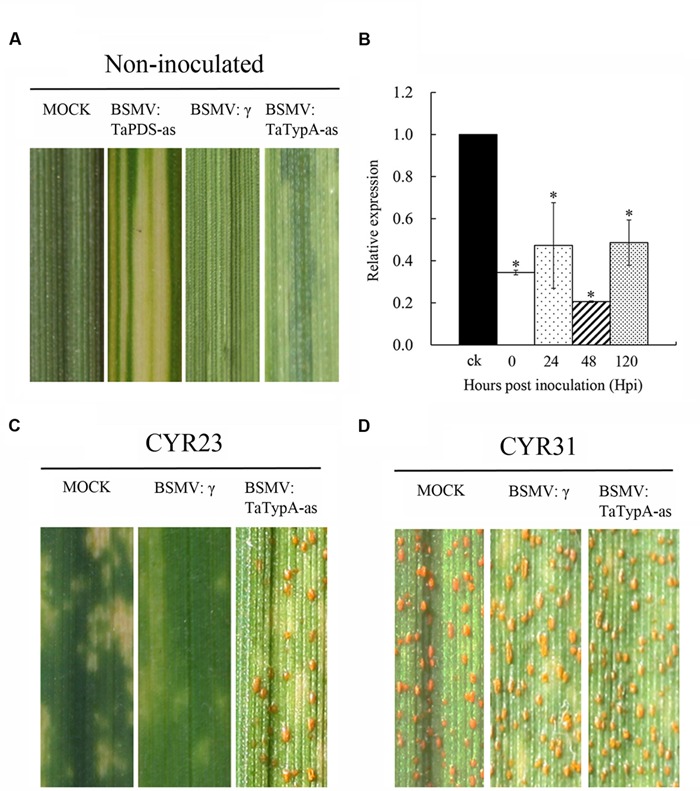
**Functional characterization of the *TaTypA* gene during interaction of wheat and *Pst* by BSMV-mediated gene silencing. (A)** Mild chlorotic mosaic symptoms on the fourth leaves of seedlings inoculated with BSMV:γ, BSMV:TaPDS-as, and BSMV:TaTypA-as at 10 dpi. Mock: wheat leaves treated with 1× Fes buffer. **(B)** Relative transcript levels of *TaTypA* in *TaTypA* knock-down plants inoculated with CYR23. The data were normalized to the *TaEF-1a* gene. CK indicates BSMV:γ. Asterisks indicate significant differences from BSMV:γ using Tukey’s HSD test (*P* < 0.05). Photographs of fourth leaves further inoculated with urediniospores of avirulent race CYR23 **(C)** or virulent race CYR31 **(D)**. Typical leaves were photographed at 15 dpi.

Virus-induced gene silencing (VIGS) is a rapid and effective reverse genetics approach in barley and wheat (Holzberg et al., 2002; Cakir and Scofield, 2008). To characterize in more detail the effect of the *TaTypA* gene in stripe rust response, BSMV-mediated VIGS was employed to silence the *TaTypA* gene(s). Su11 seedlings inoculated with BSMV:TaPDS-as exhibited strong photobleaching symptoms at 10 dpi. The presence of non-symptomatic new leaves in plants treated with Fes buffer under the same conditions indicated that silencing of *TaPDS* occurred specifically in BSMV:TaPDS-as infected plants. All wheat seedlings inoculated with BSMV: γ and BSMV:TypA-as displayed mild chlorotic mosaic symptoms on the third leaf at 10 dpi (**Figure 5A**), confirming that the BSMV-mediated gene silencing system functioned correctly and could be used in further experiments. Silencing efficiency assessed by qRT-PCR showed that *TaTypA* transcript levels were significantly reduced in *TaTypA* knock-down plants compared to control plants, with a reduction as high as 80% (**Figure 5B**). When fourth leaves of wheat plants were inoculated with the avirulent or virulent *Pst* races, an obvious HR was elicited by the avirulent CYR23 race on leaves pre-infected with BSMV:γ, BSMV:TaTypA-as and on mock-inoculated seedlings. Nevertheless, only limited fungal sporulation had occurred around the necrotic spots on leaves pre-infected with BSMV:TaTypA-as after 15 dpi compared to the other two treatments (**Figure 5C**). By contrast, wheat seedlings inoculated with virulent CYR31 showed normal disease development and numerous uredinia (**Figure 5D**). The specific fragment used in silencing is shown in **Supplementary Figure S1**. However, due to the high identity and similarity among the three copies, the silencing efficiency of each copy on A, B, and D could not be determined.

### *Pst* Growth and Host Response

**FIGURE 6:**
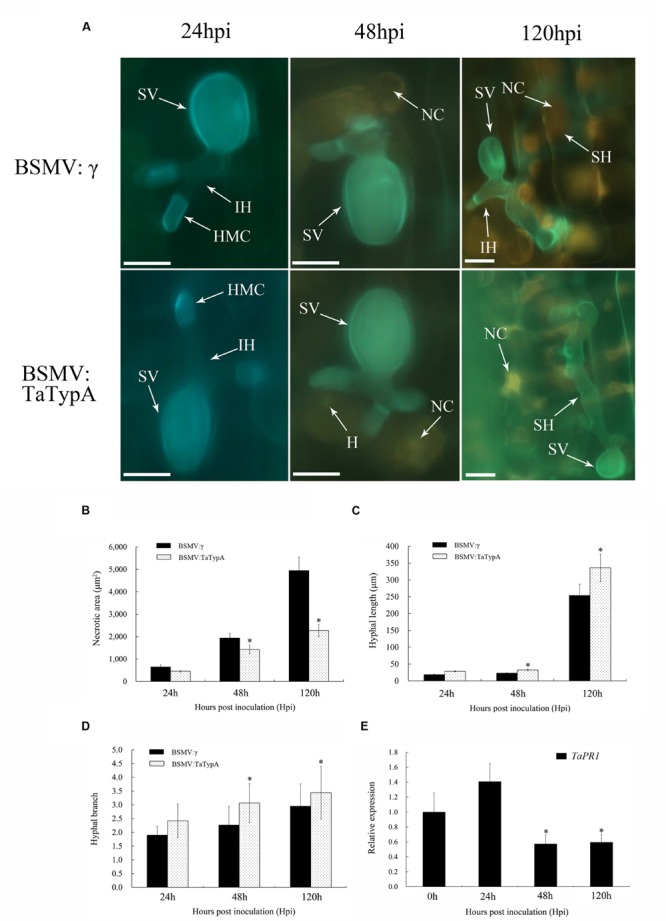
**Weakened wheat defense response in *TaTypA* knock-down plants challenged by avirulent *Pst* race CYR23.** Wheat leaves previously treated with recombinant BSMV:γ or BSMV:TaTypA were inoculated with race CYR23. **(A)** Typical leaves were examined at 24, 48 and 120 hpi. The infection sites were observed by epifluorescence. HMC, haustorial mother cell; SV, substomatal vesicle; NC, necrotic cell death; IH, primary hyphae; SH, secondary hyphae. All results were obtained from 50 infection sites. Bar, 20 μm. **(B)** Necrotic cell death was quantified as the area of autofluorescence. **(C)** Hyphal lengths are means of the average distance from the junction of the substomatal vesicle to the hyphal tip. **(D)** Hyphal branch values are means of average numbers of primary hyphae. **(E)** Expression of *TaPR1* was assayed in *TaTypA* knock-down plants compared to controls. The data were normalized to the wheat *TaEF-1a* gene. Asterisks indicate a significant differences (*P* < 0.05) from BSMV:γ using the Tukey’s HSD test.

Leaf segments from at least three plants inoculated with CYR23 were harvested from each sample to examine detailed histological changes associated with the enhanced susceptibility in *TaTypA* knock-down plants. Areas of cell death were obvious at 24, 48, and 120 hpi by microscopy and were estimated by DP-BSW software (**Figure 6A**). The areas were significantly less in *TaTypA* knock-down plants than in BSMV:γ-infected plants at 48 and 120 hpi (**Figure 6B**). There was no obvious difference in hyphal growth and branching at 24 hpi between *TaTypA* knock-down and BSMV:γ plants (**Figures 6C,D**). However, hyphal length and branch number in BSMV:TaTypA-as infected leaves were significantly (*P* < 0.05) increased relative to those in BSMV:γ infected leaves at 48 and 120 hpi, respectively (**Figures 6C,D**). The collective histological results suggest that silencing of *TaTypA* weakened the resistance of in Su11 and permitted enhanced hyphal growth and branching. The transcription level of SA-induced marker gene *TaPR1* was significantly reduced in CYR23-infected *TaTypA* knock-down plants at 48 and 120 hpi, indicating a concurrent effect on defense-related gene expression (**Figure 6E**).

### Weakened ROS Accumulation in *TaTypA* Knock-down Plants

**FIGURE 7:**
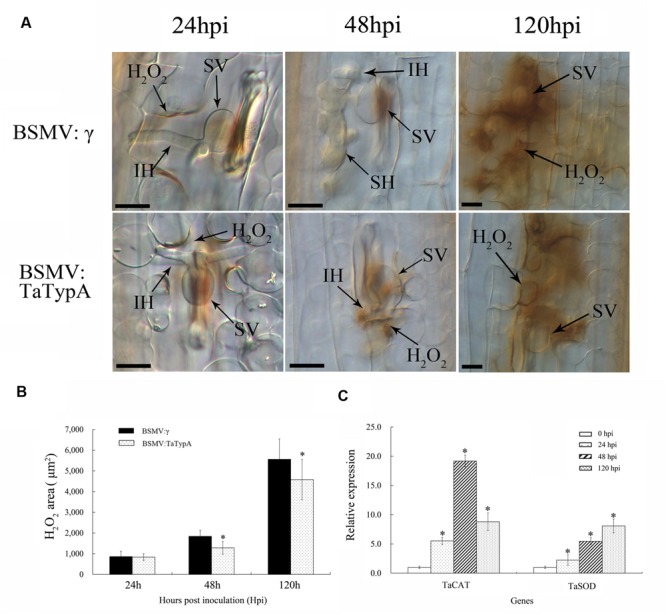
**Reduced H_2_O_2_ accumulation in *TaTypA* knock-down plants challenged by avirulent race CYR23. (A)** Wheat leaves previously treated with recombinant BSMV:γ or BSMV:TaTypA were inoculated with *Pst* race CYR23. H_2_O_2_ accumulation at infection sites was detected by staining with 3,3′-diaminobenzidine (DAB). Infection sites were observed by light microscopy. SV, substomatal vesicle; IH, initial hyphae; SH, secondary hyphae. Bar, 20 μm. **(B)** The amount of H_2_O_2_ production was measured by calculating the DAB-stained area at each infection site using DP-BSW software. Values represent mean ± standard errors of three independent assays. **(C)** Expressions of two ROS-scavenging enzyme genes were assayed in *TaTypA* knock-down plants compared to control plants. The data were normalized to wheat the *TaEF-1a* gene. Asterisks indicate significant difference (*P* < 0.05) from BSMV:γ using Tukey’s HSD test.

Reactive oxygen species accumulation with the occurrence of HR occurs in the resistance response to rust fungi in wheat (Wang et al., 2007). The induction of H_2_O_2_ by infection in *TaTypA* knock-down plants was analyzed by histochemical observation and colorimetric determination. H_2_O_2_ accumulation was significantly decreased in *TaTypA* knock-down plants compared to BSMV:γ infected plants at 48 and 120 hpi (**Figures 7A,B**). In addition, expression levels of the *TaCAT* and *TaSOD* genes involved in ROS-scavenging were significantly up-regulated in *TaTypA* knock down plants infected with CYR23 (**Figure 7C**).

## Discussion

TypA proteins are a novel class of stress tolerance-related proteins in all species ranging from prokaryotes to higher eukaryotes (Wang et al., 2008). In this study, we conducted molecular and functional analyses of wheat TaTypA in a wheat-*Pst* pathosystem.

As many as 87% of the genes in hexaploid wheat are triplicated across the A, B, and D sub-genomes (Pumphrey et al., 2009). Therefore, not unexpectedly there were three copies of *TaTypA* in the wheat genome. However, sequence alignment of three genomic copies of *TaTypA* showed that *TaTypA-6A, TaTypA-6B* and *TaTypA-6D* differed by sequence variation and deletion (**Supplementary Figure S1**). As described in previous reports, newly formed polyploids undergo a speciation process driven by combined effects of chromosomal re-patterning, gene deletion, mutation, suppression (silencing), or acquisition of modified gene function (Wendel, 2000). Thus, the differences in the three genomic copies of *TaTypA* are consistent with such events. Sequence analysis revealed that TaTypA is a typical member of the ribosome-binding GTPase superfamily, which is characterized by a ATP/GTP-binding motif, a GTP-binding elongation factor signature, and a putative ribosome-binding domain (Kiss et al., 2004). Phylogenetic analysis indicated that TypA proteins in plants form a monophyletic group, within which TaTypA and rice OsTypA constitute a subgroup (**Figure 1**). The presence of different subgroups within and between monocots and eudicots indicate possibilities for functional divergence between species and, in the case of allopolyploid plants, between sub-genomes.

In *S. salsa*, the SsTypA1-GFP fusion protein localized in the chloroplasts, and structural analysis revealed a chloroplast transit peptide sequence at the N-terminal part of the SsTypA1 protein (Wang et al., 2008). Without a chloroplast transit peptide the chloroplast localization of the protein was unexpected (Shi and Theg, 2013). In this study, we proved that the N-terminal region and the full length of TaTypA have the same subcellular localization as SsTypA1, indicating that the N-terminal region of TaTypA does contain a chloroplast transit peptide sequence. It has been confirmed that chloroplasts play a significant role in defense responses (de Torres Zabala et al., 2015). The chloroplast is a major source of ROS in plant cells and is responsible for producing the two signaling molecules required for pathogen responses, SA and jasmonic acid (Almagro et al., 2009). In addition, a few reports have shown that the resistance proteins are targeted to this organelle. For example, wheat stripe rust resistance protein WKS1 localized in chloroplasts and reduced the ability of the thylakoid-associated ascorbate peroxidase to detoxify ROS (Gou et al., 2015), and the chloroplastic/cytoplasmic protein NRIP1, which associated with the NB-LRR immune receptor “N”, was shown to be responsible for *tobacco mosaic virus* recognition and resistance in *Nicotiana* (Caplan et al., 2008). In this study, ROS accumulation was reduced during *Pst* infection in TaTypA-knock down plants. Our data suggest that TaTypA is a new chloroplast-localized defense protein, which is involved in wheat resistance to *Pst*.

Reactive oxygen species can be produced in chloroplasts through photoreaction of the herbicide MV, a superoxide anion propagator (Scarpeci et al., 2008). Wheat not only wilted when treated with certain concentrations of MV (**Supplementary Figure S4**), but transcript levels of *TaTypA* were significantly elevated in wheat seedlings exposed to MV (**Figure 3A**). This response is similar to that of *SsTypA*, which showed pronounced activation in response to H_2_O_2_ treatment (Wang et al., 2008). ROS, such as superoxide, hydrogen peroxide, and hydroxyl radicals, were induced by drought, salt, and cold stress. The increased expression of *TaTypA* in response to cold stress (**Figure 3B**) may result from the excessive ROS generated. However, the transcript level of *TaTypA* was not significantly changed under salt or drought stress. As previous studies showed, the biphasic production of ROS consists of a primary phase that occurs within minutes and a secondary phase that occurs within hours/days under abiotic stress (Mittler et al., 2011). It seems that *TaTypA* was not involved in primary phase of ROS production under drought and cold stress. On the contrary, *SsTypA1* was induced by NaCl and mannitol (drought stress) treatments as early as 6 and 4 hpt, respectively (Wang et al., 2008). These differences in expression of *TaTypA* and *SsTypA1* may be associated with divergence of monocots and dicots or different treatment methods used. Different plant species are highly variable in response to environmental factors; an environmental condition that is harmful to one species may not be stressful for another (Munns and Tester, 2008). This is also evidenced by the multitude of different stress-response mechanisms. Hence, we propose that the functions of *TaTypA* and *SsTypA1* differ when plants are subjected to the same environmental stresses.

TypA/BipA proteins are widely distributed in all organisms. However, there has been little research on the functions of TypA proteins in plant disease responses. Our results in the present study indicated that *TaTypA* positively regulates the response of wheat to *Pst*. Silencing of *TaTypA* reduced the level of race-specific immunity to *Pst* in *TaTypA* knock-down plants. In response to the avirulent *Pst* race CY23, *TaTypA* knock-down Su11 wheat plants expressed weakened resistance characterized by decreased necrotic area, reduced ROS accumulation and increased uredinia production. Previous histological and cytological observations revealed oxidative bursts during the early (12–24 h) and late (96–120 h) stages of infection in incompatible interactions between wheat and *Pst* (Wang et al., 2007). In contrast, the hyphae of *Pst* rapidly colonized host tissues during the compatible interaction without any detectable O_2_^⋅-^ and H_2_O_2_ accumulation or HR. *TaTypA* expression was induced as early as 36 hpi and peaked at 96 hpi in the incompatible interaction, suggesting that *TaTypA* may be responsible for ROS accumulation and protection of host cells from *Pst* infection. Thus, silencing of *TaTypA* led to decreased ROS accumulation in *TaTypA*-knock-down plants. Superoxide dismutase (SOD) and catalase (CAT) are major ROS-scavenging enzymes (Mittler et al., 2004). H_2_O_2_ can be eliminated by increased catalase and superoxidase activities. The accumulation of *TaSOD* and *TaCAT* in *TaTypA* knock-down plants was induced by CYR23 infection, suggesting that *TaTypA* is an important factor in the ROS-scavenging system. Decreased catalase and ascorbate peroxidase activities in tobacco resulted in plants with increased resistance to pathogens (Mittler et al., 1999), whereas overexpression of catalase resulted in plants that were more susceptible (Polidoros et al., 2001). These results suggest that the ROS-scavenging systems have an important role in managing ROS generated in response to pathogens. Our results suggest that *TaTypA* positively regulates resistance to stripe rust in a ROS-dependent manner.

Hyphal length and number of branches are measures the ability of the pathogen to colonize the host under the stress imposed by HR at infection sites. The necrotic area triggered by infection of avirulent race CYR23 was used to represent the level of the HR, which is probably triggered by generation of ROS (Wang et al., 2007; Zurbriggen et al., 2010). In *TaTypA* knock-down plants, ROS accumulation was reduced in CYR23 infection at 48 and 120 hpi, coinciding with decreased hyphal length, necrotic area and hyphal branch number, suggesting that increased colonizing ability of the pathogen is a consequence of decreased host resistance. ROS are the well-characterized second messengers in a variety of cellular processes in plants, including tolerance to environmental stress (Desikan et al., 2001). ROS has been shown to regulate different plant hormone signaling pathways, plant-biotic interactions and developmental processes by redox-dependent regulation of transcription factors (Barna et al., 2012). One of the most thoroughly characterized defense-signaling pathways regulated by oxidation events is the induction of salicylic acid (SA)-dependent responses (Fu and Dong, 2013). In the present study, *TaPR1*, the marker gene of the SA pathway, was significantly decreased by CYR23 infection at 48 and 120 hpi. We infer that *TaTypA*, functions in the SA-signaling pathway via ROS-dependent signals and thereby contributes to systemic acquired resistance.

To our knowledge this is the first report to confirm that *TypA* functions in biotic stress. Knock-down of *TaTypA* demonstrated its positive role in regulation of hypersensitive cell death and in resistance of wheat to stripe rust. On the basis of our results, we believe that TypA has an important role in plant disease resistance, but determination of the precise functional mechanism of *TaTypA* requires further investigation.

## Author Contributions

JG and ZK designed the experiment. PL, TM, WM, DL, TQ, and JG performed the experiments and analyzed the data. PL, TM, JG, and ZK wrote the manuscript.

## Conflict of Interest Statement

The authors declare that the research was conducted in the absence of any commercial or financial relationships that could be construed as a potential conflict of interest.

## Footnotes

1 http://wheat-urgi.versailles.inra.fr/Seq-Repository/BLAST

2 http://www.ncbi.nlm.nih.gov/gorf/gorf.html

3 http://www.ncbi.nlm.nih.gov/blast/

4 http://pfam.xfam.org/

5 http://www.ebi.ac.uk/interpro/search/sequence-search

6 http://prosite.expasy.org/scanprosite/

## Supplementary Material

FIGURE S1 **Multi-alignment of the encoding sequence of the three copies of *TaTypA*.** *TaTypA* cDNA, the full-length cDNA sequence amplified from cDNA of wheat cv. Suwon 11. The cDNA sequences of three *TaTypA* copies from chromosomes 6AL, 6BL, and 6DL were obtained from the wheat UGRI genome database of wheat cv Chinese Spring. The red and blue boxes indicate the initiation codon (ATG) and termination codon (TAG), respectively.

FIGURE S2 **Multi-alignment of the deduced TaTypA proteins from wheat cv. Suwon11 and Chinese Spring genome database.** Identical and similar amino acid residues are shaded in black and light gray, respectively.

FIGURE S3 **Multi-sequence alignment of *TaTypA* against TypA from other species.** Identical and similar amino acid residues are shaded in black and light gray, respectively. Motifs indicated in the sequence: (I) ATP/GTP binding site; (II) GTP-binding elongation factor signature; (III) putative ribosome binding domain. Triangles indicate conserved tyrosine residues in all nine proteins. Ta, *Triticum aestivum*; Bd, *Brachypodium distachyon*; Os, *Oryza sativa*; Zm, *Zea mays*; Sb, *Sorghum bicolor*; Gm, *Glycine max*; Ss, *Suaeda salsa*; At, *Arabidopsis thaliana*; Tp, *Trifolium pretense*.

FIGURE S4 **The expression of GFP, TaTypA::GFP and TaTypA-N_1-80_::GFP proteins was determined by Western blot analysis using GFP antibody.** The GFP antibody was diluted in blocking buffer at 1:5000. The graph shows the relative band using image Lab 3.0 software.

FIGURE S5 **Symptoms of wheat seedlings treated with MV. Ten-days-old wheat seedlings were sprayed with 0.1, 1, and 5 mM MV under the normal growing conditions.** CK, wheat seedlings sprayed with water.

TABLE S1 **Primers used for TaTypA analyses**.
