# Supplementary figures and images for: TaTypA, a Ribosome-Binding GTPase Protein, Positively Regulates Wheat Resistance to the Stripe Rust Fungus
Source: Front Plant Sci. 2016 Jun 21;7:873. doi: 10.3389/fpls.2016.00873 (PMC4914568; doi:10.3389/fpls.2016.00873)

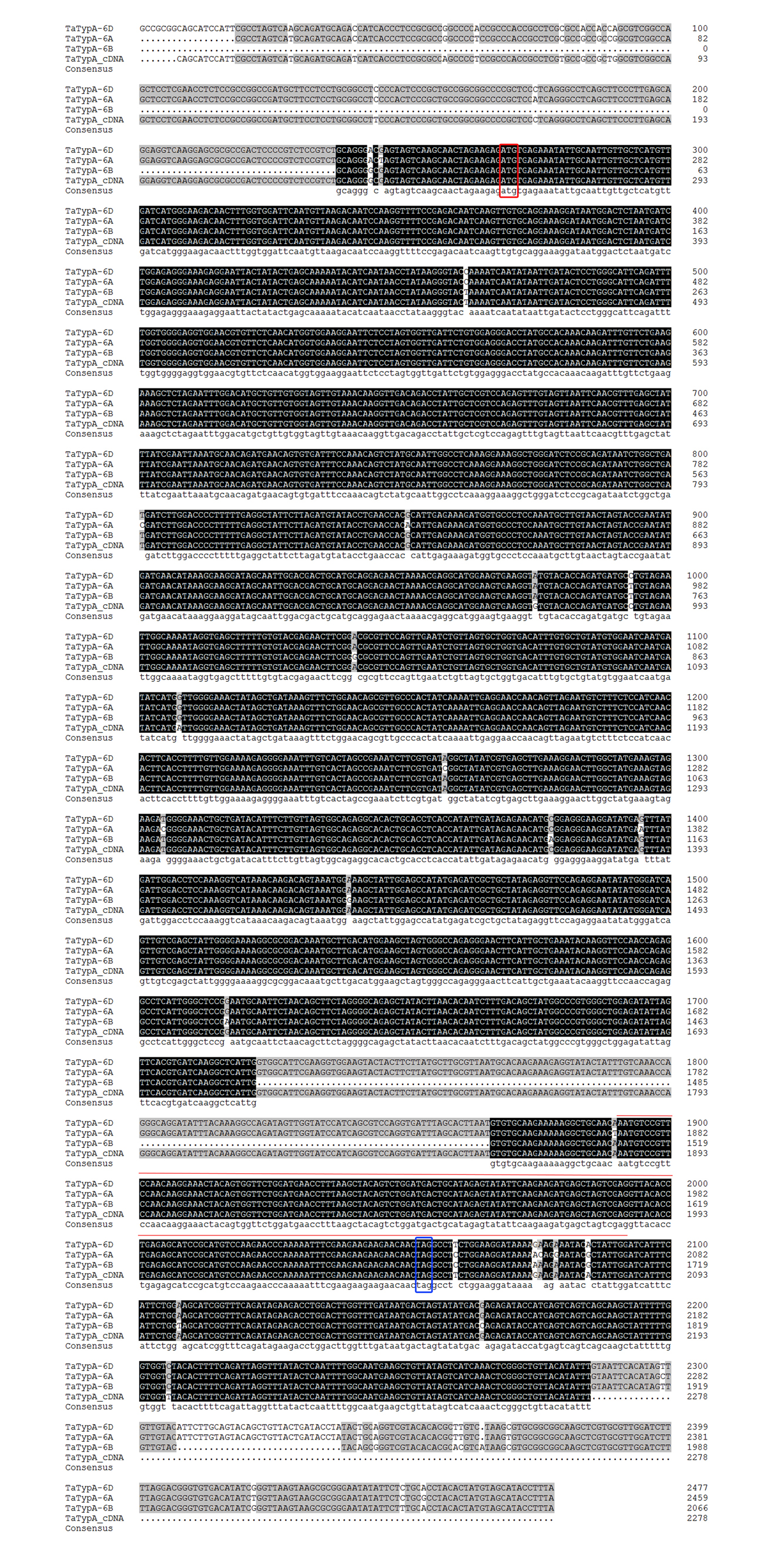

Supplement: FIGURE S1 — Multi-alignment of the encoding sequence of the three copies of TaTypA. TaTypA cDNA, the full-length cDNA sequence amplified from cDNA of wheat cv. Suwon 11. The cDNA sequences of three TaTypA copies from chromosomes 6AL, 6BL, and 6DL were obtained from the wheat UGRI genome database of wheat cv Chinese Spring. The red and blue boxes indicate the initiation codon (ATG) and termination codon (TAG), respectively. [file Image_1.JPEG]

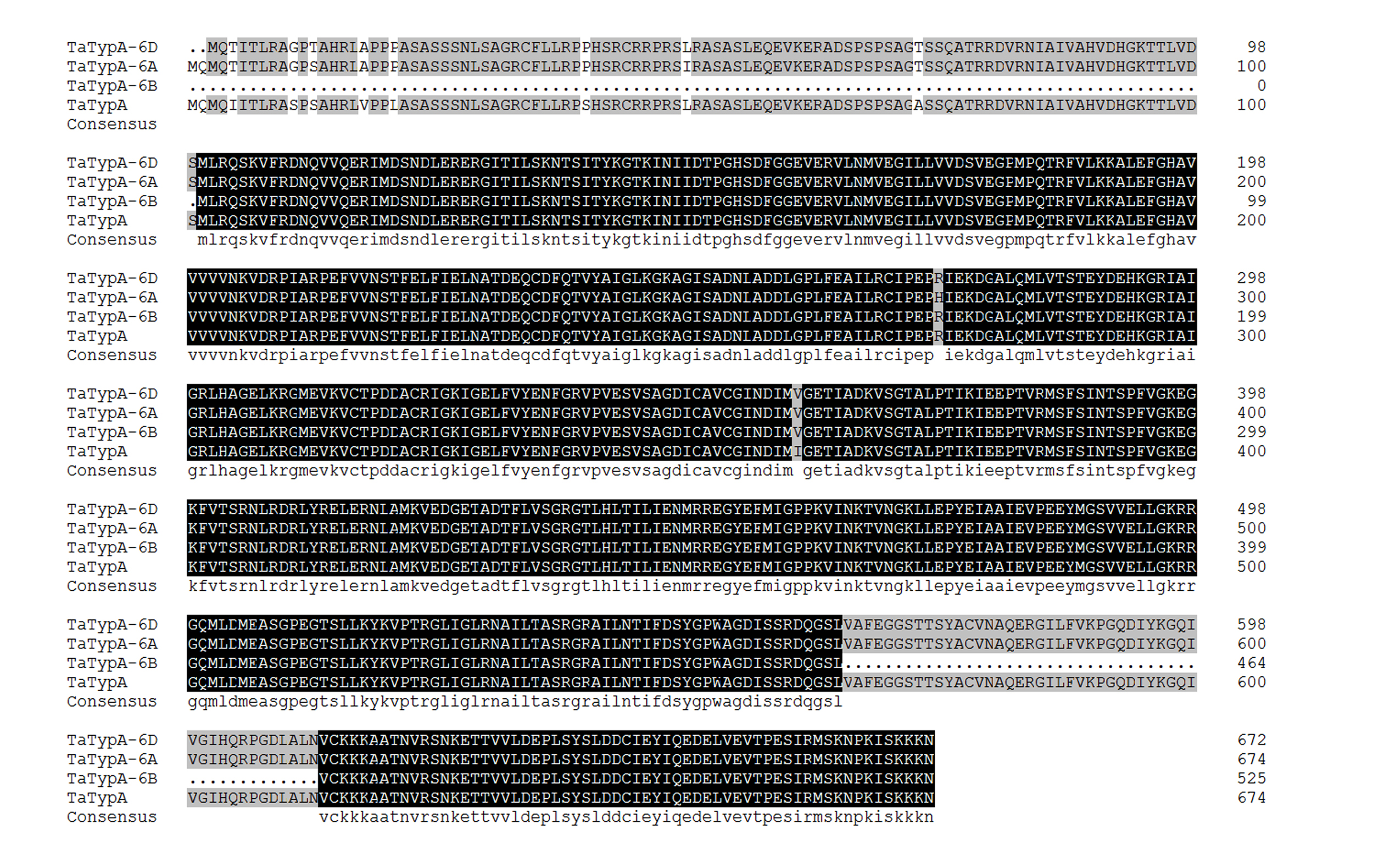

Supplement: FIGURE S2 — Multi-alignment of the deduced TaTypA proteins from wheat cv. Suwon11 and Chinese Spring genome database. Identical and similar amino acid residues are shaded in black and light gray, respectively. [file Image_2.JPEG]

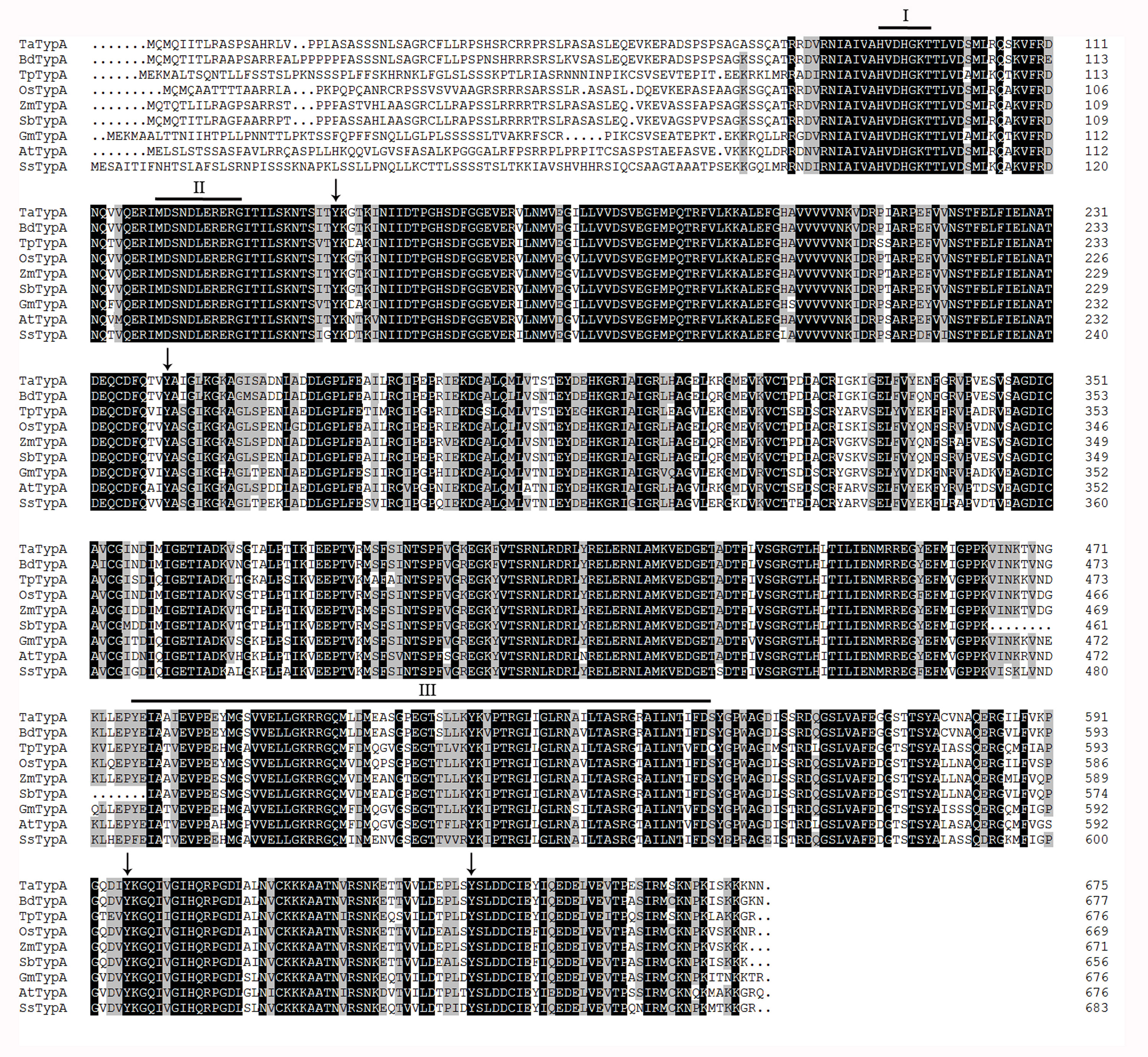

Supplement: FIGURE S3 — Multi-sequence alignment of TaTypA against TypA from other species. Identical and similar amino acid residues are shaded in black and light gray, respectively. Motifs indicated in the sequence: (I) ATP/GTP binding site; (II) GTP-binding elongation factor signature; (III) putative ribosome binding domain. Triangles indicate conserved tyrosine residues in all nine proteins. Ta, Triticum aestivum; Bd, Brachypodium distachyon; Os, Oryza sativa; Zm, Zea mays; Sb, Sorghum bicolor; Gm, Glycine max; Ss, Suaeda salsa; At, Arabidopsis thaliana; Tp, Trifolium pretense. [file Image_3.JPEG]

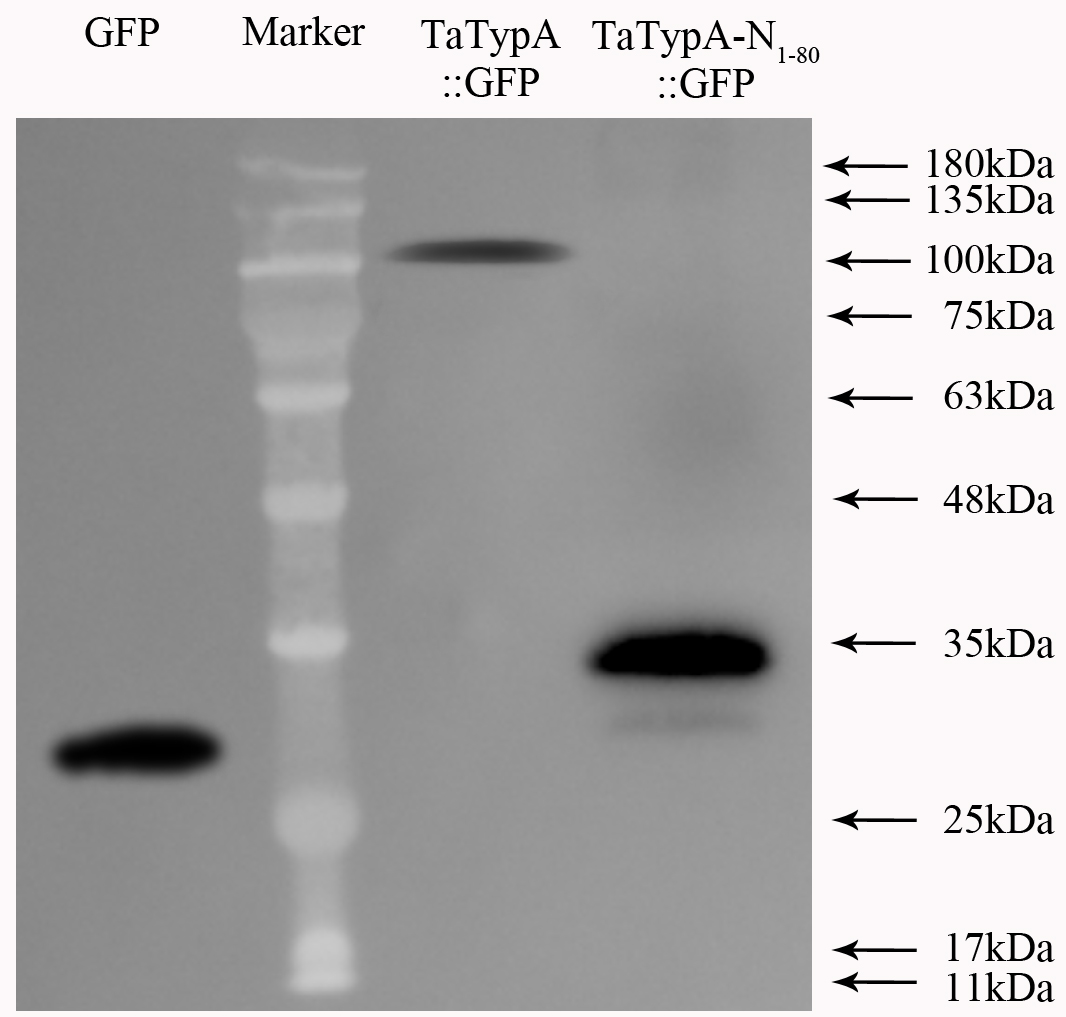

Supplement: FIGURE S4 — The expression of GFP, TaTypA::GFP and TaTypA-N1-80::GFP proteins was determined by Western blot analysis using GFP antibody. The GFP antibody was diluted in blocking buffer at 1:5000. The graph shows the relative band using image Lab 3.0 software. [file Image_4.JPEG]

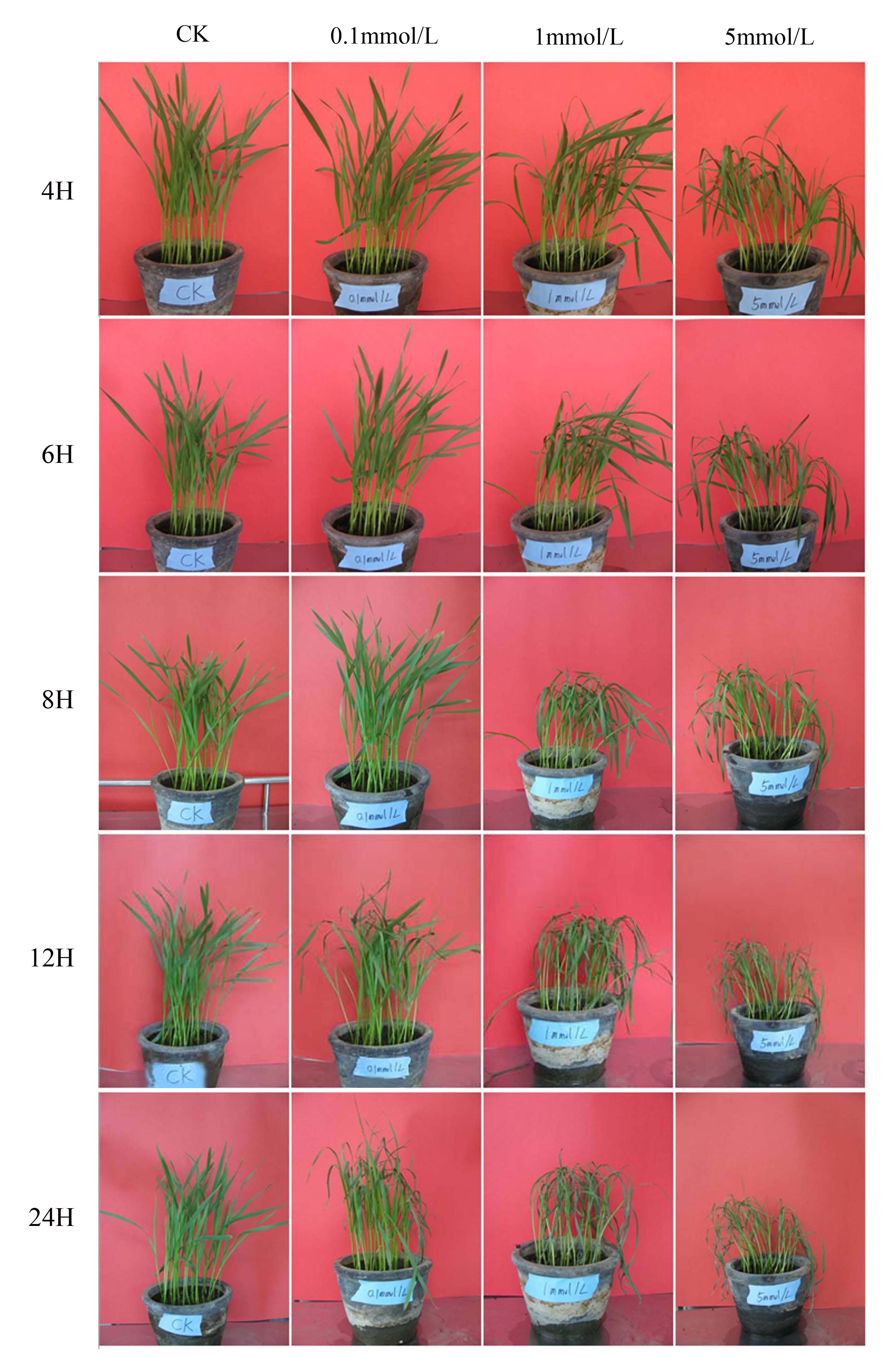

Supplement: FIGURE S5 — Symptoms of wheat seedlings treated with MV. Ten-days-old wheat seedlings were sprayed with 0.1, 1, and 5 mM MV under the normal growing conditions. CK, wheat seedlings sprayed with water. [file Image_5.JPEG]
